# Age Is Not a Limiting Factor in Interventional Radiotherapy (Brachytherapy) for Patients with Localized Cancer

**DOI:** 10.1155/2018/2178469

**Published:** 2018-01-21

**Authors:** Valentina Lancellotta, György Kovács, Luca Tagliaferri, Elisabetta Perrucci, Giuseppe Colloca, Vincenzo Valentini, Cynthia Aristei

**Affiliations:** ^1^Radiation Oncology Section, Department of Surgery and Biomedical Sciences, University of Perugia and Perugia General Hospital, Perugia, Italy; ^2^Interdisciplinary Brachytherapy Unit, University of Lübeck/UKSH-CL, Lübeck, Germany; ^3^Polo Scienze Oncologiche ed Ematologiche, Università Cattolica del Sacro Cuore, Fondazione Policlinico Universitario Agostino Gemelli, Roma, Italy; ^4^Radiation Oncology Section, Perugia General Hospital, Perugia, Italy; ^5^Polo Scienze Oncologiche ed Ematologiche, Università Cattolica del Sacro Cuore, Fondazione Policlinico Universitario Agostino Gemelli, Gruppo Italiano di Oncologia Geriatrica (GIOGER), Roma, Italy; ^6^Polo Scienze Oncologiche ed Ematologiche, Istituto di Radiologia, Università Cattolica del Sacro Cuore, Fondazione Policlinico Universitario Agostino Gemelli, Roma, Italy

## Abstract

This review examines the role of interventional radiotherapy (IRT otherwise known as brachytherapy) in cancer treatment for elderly patients. Despite their advanced age and associated comorbidities, elderly patients should receive definitive cancer therapies, including surgery and radiotherapy (RT). In fact, RT becomes first-line option for patients who are not eligible for surgery (due to comorbidities, anticoagulant drugs, and risk of disfigurement) or those who refuse it. It emerged from this review of the literature as effective, simple, safe, and comfortable and was associated with good local control, low toxicity rates, and excellent cosmesis and provided a cost benefit. IRT may be used as sole treatment for small cancers or as a useful adjunct to surgery or external beam radiotherapy (EBRT) in more advanced (or lymph node positive) cases, especially when the aim is local control with adequate preservation of normal tissue function. As palliative treatment, IRT preserves quality of life and/or improves survival. It is to be hoped that this review will serve as a helpful guide for members of multidisciplinary teams that are involved in treating elderly patients with cancer.

## 1. Introduction

The National Institute on Aging described aging in our society as a “silver tsunami for which we are unprepared.” Being a complex multidimensional, dynamic process deriving from interactions among a range of environmental and genetic factors, aging leads to loss of functional reserve and organ system susceptibility to gradual deterioration, that is, “frailty” [1–3]. Since over 50% of tumors are currently diagnosed in patients aged 65 years or more and the rate is expected to rise to 70% by 2030, the management of elderly cancer patients is a major issue for health authorities. This is particularly worth noting since they constitute a heterogeneous group due to differences in lesion locations, multimorbidities, physical or functional performance status, social support, and physiological age-related changes, which may make surgery, which is often the first-line option for many cancers, not feasible in this advanced age group [4].

When assessing the elderly and adapting treatment to them, clinicians should focus on identifying frail or prefrail patients as age-related status determines their ability to tolerate and comply with treatment, their survival, and quality of life [5, 6]. These factors may also be associated with delayed diagnosis and in fact a significant relationship emerged between age and disease stage at diagnosis in different tumor sites [7]. On the other hand, the biological aggressiveness of tumors in aged patients also seems to vary with the individual's age, being lower for some histological types in older patients [8–10]. Indeed, some clinical studies concluded that, for selected patients, the age factor does not decrease tolerance to more aggressive treatments [11–13].

Even though RT indications in elderly cancer patients have long been debated, at present age alone no longer constitutes a counter-indication and if the aim of radiotherapy is curative, there is no age-related indication for a dose reduction [14, 15]. The modern consensus is that elderly cancer patients should receive RT, if their performance status is adequate and the extent and severity of comorbidities do not preclude it.

Consequently, RT is often treatment of choice for the aged, and IRT, which was less likely to be delivered for patients ≥80 years, may be considered competitive, when compared with other radiotherapy modalities or cancer treatments [16]. The American Brachytherapy Society (ABS) recently published a consensus statement that provides detailed technical guidance for IRT for medically inoperable cancer [2], with emphasis on IRT with or without EBRT for most patients. The Radiotherapy Task Force of the International Society for Geriatric Oncology (SIOG) provided recommendations for RT in elderly patients without focusing on IRT, which offers several advantages over external beam techniques [17]. Even though IRT delivery often requires anesthesia, particularly in perioperative or interstitial settings, treatment times are shorter, less normal tissue is irradiated, and gating techniques are not needed to counteract patient movement. Local dose applications using different radiotherapy techniques are compared in Table 1. The following review focuses on IRT applications in this growing cohort of elderly patients and summarizes the evidence for offering it to them, considering that IRT was associated with acceptable toxicity rates in the elderly and that some types of acute side effects are observed more frequently in younger patients [18].

## 2. Material and Methods

On 12 April and 17 July 2017, MEDLINE searches with* “brachytherapy”* and* “elderly”* as key words were performed in the* Knowledge Finder*® and* Scopus*® databases for the years 2012–2017.* Knowledge Finder* yielded 24 publications, 13 of which were relevant.* Scopus* provided 387 reports, 26 of which were relevant. Pertinent reports encompassed the following categories: general (*n* = 3), anal and rectum (*n* = 4), bile duct (*n* = 2), uterine cervix (*n* = 2), uterine corpus (*n* = 1), vaginal and vulvar (*n* = 1), head and neck (*n* = 6), esophagus (*n* = 1), lung (*n* = 1), prostate (*n* = 4), skin (*n* = 2), and breast (*n* = 2) respectively. All reports were studied and analyzed.

## 3. Results and Discussion

IRT is used alone or as a boost to external beam RT (depending on tumor site and the patient's condition) [19, 20]. IRT alone is associated with excellent outcomes as local control, patient survival rates, cosmetic results, and the incidence and severity of side effects did not differ in the aged and in younger populations [21–25]. Although the combination therapy (EBRT + IRT) is associated with the highest overall survival (OS) rate, nearly 40% of patients do not receive either surgery or radiotherapy and are treated with systemic therapy alone (e.g., endocrine therapy). The incidence of late toxicity ranged from 0% to 21%. After high dose-rate- (HDR-) IRT alone or in combination with EBRT, Disease Specific Survival (DSS) and low complication rates, with no grade 3 or higher acute toxicities, were similar in patients aged 75–92 years (median 83 years) and other EBRT studies [26].

### 3.1. Anal and Rectal Cancer

Because of potential complications or poor organ function, patients aged 70 years or more are not usually considered as suitable candidates for surgery or radiochemotherapy. Although they were often treated with palliative RT, they might benefit from a more radical approach using RT alone [27]. Promising results were achieved with contact X-ray RT, which delivers high doses to the tumor surface, in combination with EBRT in inoperable patients [28–31]. An alternative to contact X-ray for inoperable patients is HDR endorectal IRT [32, 33] which was assessed in association with EBRT in a few retrospective studies [34, 35]. No agreement emerged on the optimal dose, toxicity profile, or treatment schedules. Despite these limitations, all studies reported a response rate of almost 90%, with approximately 60% complete remission (CR) and a good local progression-free survival rate (L-PFS) in complete responders [28–33, 36]. IRT alone or as a boost to EBRT (+/− chemotherapy) also provided excellent results [37–39]. Furthermore, the toxicity rate was acceptable in all reports and anal function was preserved. The latter parameter is one of the major objectives of anal and rectal cancer therapy because colostomy is a major impairment to quality of life and should always be avoided, if possible.

### 3.2. Bile Duct Cancer

As extrahepatic biliary carcinoma (EBC) is rare, accounting for about 2–6% of all cancer [40], few data can be extrapolated as referring specifically to the aging population.

Although endoscopic stenting is first-line palliative therapy, stent occlusions rates of 30–45% were reported, as palliative treatment EBRT alone prolongs survival and reduces symptoms but only with major local dose escalation which is, unfortunately, limited by the tolerance of critical organs like the liver, kidneys, and small bowel.

Intraluminal IRT (ILIRT), an optimal technique for delivering a high dose to a well-defined small volume and the shortest possible treatment time, has the further advantage of respecting the dose constraints of organs at risk. In a retrospective comparison of 30 patients treated with self-expanding metallic stent (SEMS) + ILIRT versus SEMS alone, Karani et al. reported the combined approach was associated with longer prolongation of biliary patency and up to 16.8 months longer mean OS [41]. Válek et al. randomized 42 patients to either palliative stent placement or stent placement with ILIRT followed by EBRT, reporting that OS was significantly increased in patients who received ILIRT [42]. OS rates at 6, 12, and 18 months after SEMS + ILIRT in 18 patients with nonmetastatic extrahepatic biliary cancer (median age 79 years, range 61–86) were 77%, 59%, and 37%, respectively. Only one patient developed G3 gastrointestinal toxicity [43].

Consequently, we agree that bile duct ILIRT should be treatment of choice in some cases, depending on lesion location [40] because it plays a limited, but specific role, in curing early disease, has a place in the postoperative treatment of small residual disease, and is useful as palliative therapy.

### 3.3. Gynecological Cancer

#### 3.3.1. Cervix Cancer

Although radiochemotherapy including IRT is standard treatment for locally advanced or inoperable cervical cancer, data are based on studies with the majority of the patients under 70 years [44, 45] and few data are available about treatments, toxicities, and outcomes in elderly women. The National Cancer Institute's Surveillance, Epidemiology, and End Results (SEER) database contains data on 28902 patients with cervical cancer but only 13.5% were ≥70 years old. Elderly women usually suffered from squamous cell tumors, G3, and advanced stage disease [6] and were treated differently from younger patients [46–49]. After surgery for early stage disease, elderly patients were less likely to receive curative or adjuvant radiotherapy, even when there was an indication. For example, 20% aged 70–79 years and 40% aged >80 years did not receive IRT [50].

Lin et al. recruited 126 patients with cervical cancer (median age 81.5 years; range 75–98) who underwent curative (81 received definitive RT, 10 adjuvant RT) or palliative RT [51]. Overall, 3- and 5-year OS rates were, respectively, 52.7% (95% CI, 43–61) and 41.2% (95% CI, 32–49), while Recurrence-Free Survival (RFS) rates were 62.88% (95% CI 53–70) and 59.51% (95% CI, 49–67), respectively. In 91 patients who received curative RT, 3- and 5-year OS rates were, respectively, 66.63% (95% CI, 55–75) and 54.48% (95% CI, 43–64), while RFS rates were 75.86% (95% CI, 65–83) and 71.57% (95% CI, 60–80), respectively. G3 gastrointestinal toxicity was observed in 7% of patients. No G3 bladder and vaginal toxicity was reported.

In underlining the importance of individualized treatment, Chen et al. [52] included IRT in the treatment regimen of elderly patients with cervical cancer. Adapting the IRT schedule to the patient's general condition ensures the shortest possible total treatment time. In comparing outcomes in the 70 and 80 age groups, Yanazume et al. stated that incomplete intracavitary IRT significantly decreased survival rates [53].

#### 3.3.2. Endometrial Cancer

It occurs mainly in elderly women with a median age of 68 years at diagnosis who usually presented with unfavorable prognostic factors like nonendometrial histology, advanced stage, G3, and more aggressive immunological characteristics [54–57]. They have a higher recurrence rate and cancer-specific mortality than younger women and are often undertreated [58–60]. Lymphadenectomy, RT, and systemic treatment are less likely to be administered to older women [57, 61–63]. Lymphadenectomy was not recommended for 58.2% of patients aged 71–80 years versus 30.8% of those aged ≥81 years, respectively. RT was not recommended for 76.6% versus 51% of cases and systemic therapy was not recommended for 58.6% versus 25% [64]. Elderly patients did not have more perioperative complications than younger patients but it should be borne in mind that laparoscopy was associated with less morbidity than laparotomy in terms of sedation time, blood loss, and perioperative complications [65–68]. After surgery, pelvic/vaginal relapse rates were higher in patients who did not receive adjuvant RT [69].

RT provided good local control and low toxicity and improves OS, particularly when EBRT was combined with IRT [70]. Indeed, 10-year DSS and OS rates were, respectively, 75.1% and 30.2%, in 228 patients with stage I endometrial cancer who were treated with IRT alone [71]. Furthermore, the American Brachytherapy Society published a consensus statement with emphasis on IRT with or without EBRT for inoperable endometrial cancer. They recommended IRT alone for inoperable, clinical stage I endometrial cancer when there was no MRI evidence of lymph node involvement or deep myometrium invasion [72]. Decision-making, however, needs to balance risk factors and the patient's health status, independently of age. In assessing the impact of adjuvant radiotherapy (EBRT +/− IRT) in terms of feasibility and activity in women aged ≥75 years, Fiorica et al. observed survival was significantly better in patients with no, or only mild, comorbidities and concluded that aggressive treatment risked shortening life expectancy in patients with severe comorbidities [73]. Indeed, the right treatment schedule for them could well be conservative surgery combined with postoperative intravaginal IRT.

#### 3.3.3. Vulva and Primary Vagina Cancer

The rare vaginal and vulvar cancer account for only 4% of gynecological malignancies. Known risk factors are age, smoking, human papilloma virus, or human immunodeficiency virus infection as well as other genital cancers. The average age at diagnosis of invasive vulvar cancer is 70 years and 50% of vaginal cancers occur in women aged ≥70 years [72]. Since most elderly patients have multiple comorbidities that often restrict treatment options [74], locally advanced or recurrent vulvar cancer may become a serious treatment issue.

IRT as monotherapy or in association with EBRT [75–83] can exert local control in the majority of patients with acceptable morbidity rates. Results with multimodality treatment varied with cancer stage: 86% of patients with stage I/II vulvar cancer achieved DFS at 5 years, which dropped to 54% for stage III/IVA and 16% for stage IVB [84]. In vaginal cancer 5-year DFS rates were, respectively, 80–90%, 35–78%, 30–59%, and 0–58% for stages I, II, III, and IV. After interstitial IRT, patients exhibited milder but similar rates of late mucosal reactions and more severe stenosis than after intracavitary IRT [85].

### 3.4. Head and Neck Cancer

IRT offers excellent long-term results as local treatment of head and neck (H&N) cancers [86] in “elderly patients” with the cut-off being usually set at age 65 years [87]. Even though advanced age usually precludes elective neck surgery or RT, it should be considered for locally advanced N0 cases. If even node dissection is not performed the relapse risk rises to over 20% [88].

Performance status is the major factor in treatment tolerance, followed by tumor stage, anatomic site, and oral hygiene status. Even though surgery is first-line treatment of choice if the tumor is resectable, a satisfactory surgical margin may be associated with significant loss of function and/or cosmetic compromises, which could potentially be overcome by combining surgery with perioperative IRT [89]. In fact, in resected H&N cancer, the complication rates, locoregional failure, and disease-free survival were similar to limited-volume perioperative HDR-IRT and wide-field EBRT, despite a much smaller treatment volume. Careful delineation of the areas at high risk of recurrence may provide a useful basis for volume reduction and improve the therapeutic ratio [90].

After IRT treatments for primary irradiated oral cavity and oropharynx cancers, Yuasa-Nakagawa et al. reported DSS and OS rates of 63% and 49%, respectively [91]. No significant differences emerged in 2- and 5-year local control rates of node negative tongue cancer when very elderly (≥80 years) patients were compared with younger counterparts (91% versus 82%). The included intercurrent deaths OS rates were 55% at 2 years and 34% at 5 years [92]. On the other hand, 7 years after IRT for tongue cancer Khalilur et al. observed 70% DSS rates in patients ≥80 years compared with 41% in the cohort ≤80 [93]. HDR-IRT provided excellent results in a large cohort of patients with lip cancer, as local control rates were 100% in T1, 93.9% in T2, and 80% in T4 cancers. Unfortunately no information was available on age-related outcomes in this study [94].

Outcomes varied after nasopharyngeal cancer treatment in the elderly compared with the general adult population. Zhang et al. reported that EBRT +/− IRT boost might inherently predict poor outcomes in elderly patients, as the cure rate correlated strongly with the applied dose. Unfortunately, the cohort size precluded comparing different boost methods at the same dose levels [95].

### 3.5. Esophagus Cancer

Although esophagus cancer occurs in 19.6% of patients aged ≥75 years [96, 97], elderly patients underwent less surgery, radiotherapy, and chemotherapy than younger cohorts. Patient's age, lymph node-status, and radiation dose determine the optimal individualized treatment for the elderly. The RTOG 8501 study showed platinum-based chemotherapy plus EBRT benefited OS more than EBRT alone. EBRT + IRT provided good outcomes in locally advanced esophageal squamous cell cancer [98]. In a retrospective cohort study of 191 patients (median age 75 years, range 70–84), Li et al. reported 1-, 2-, 3-, and 5-year OS rates of 68.5%, 48.2%, 40.3%, and 28.7%, respectively [99] with 1-, 2-, 3-, and 5-year rates for locoregional control of 82.2%, 67%, 61.8%, and 54.2%, respectively. EBRT + IRT appeared to be efficacious, with acceptable toxicity levels as there were no perforations, 33.5% G2 esophagitis, 2.6% fistula, and 7.9% massive bleeding. HDR-IRT alone or in combination with EBRT is also excellent for palliation of pain, dysphagia, or obstruction. After randomizing 41 patients to receive SEMS placement followed by HDR-IRT or HDR-IRT alone, Amdal et al. concluded that combination treatment with SEMS and HDR-IRT may be indicated for patients needing immediate dysphagia relief, while HDR-IRT alone may be indicated for less urgent cases as it was associated with a lower rate of complications [100].

### 3.6. Lung Cancer

IRT may be applied as a curative/palliative monotherapy in elderly patients with lung cancer. ILIRT is optimal for treating intraluminal obstruction due to tumor infiltration which is one of the major symptoms of lung cancer and may be combined with stenting and/or laser resection. It is rarely used intraoperatively after wedge resection with either implantation of plastic tube applicators for HDR-IRT or low dose-rate (LDR) isotopes. Response rates of 97% for hemoptysis, 93% for dyspnea, 91% for obstructive pneumonia, and 82% for cough were reported, respectively [101]. McKenna et al. reported on the combination of wedge resection and interstitial perioperative IRT in 48 patients with poor pulmonary function who underwent wedge resection, node dissection, and HDR-IRT with a dose of 7x 3.5 Gy in 4 treatment days. After a 13.5-month follow-up, three local recurrences were observed [102]. Santos et al. compared wedge resection versus wedge resection and intraoperative I-125 seed implantation in cohorts of 101 and 102 stage I non-small-cell lung cancer (NSCLC) cases. No significant differences emerged in morbidity, mortality, and survival but the implanted group had significantly fewer local recurrences (2% versus 19%, *p* = 0.0001) [103]. Furthermore, after one cycle of first-line chemotherapy and CT guided I-125 seed implantation in locally advanced NSCLC, Song et al. reported survival and quality of life were significantly better than with best supportive care [104].

Combining EBRT + ILIRT, however, seems to be meaningful only if large, intraluminal tumors remain after completing EBRT. In a meta-analysis of 13 randomized trials, EBRT + ILIRT did not improve symptom control or OS compared with EBRT alone [105]. Similarly, a meta-analysis of 29 trials of endobronchial IRT + EBRT in NSCLC found it to be more effective for symptom relief than EBRT alone [106].

### 3.7. Prostate Cancer

Because of the rise in the aging population, interest is currently focusing on the increased incidence of prostate cancer. Age is the greatest risk factor since autopsy studies have found prostate cancer in 30% men ≥50 years [107]. As the elderly undergo fewer check-ups than younger men [108], prostate cancer may already have metastasized at the time of diagnosis.

HDR and LDR IRT play central roles at all stages of prostate cancer treatment. They are used as monotherapy in localized disease, as complementary to EBRT (+/− antiandrogen treatment), or as a salvage treatment for local recurrences [109].

Choice of treatment in localized or locally advanced prostate cancer needs to be based on risk categories [110]. Cooperberg et al. described the danger of overtreatment in the elderly [111]. In 4,4630 men aged 65 to 80 years, Wong et al. reported better survival rates with active treatment rather than watchful waiting for low- and intermediate-risk subgroups [112].

HDR-IRT is established as successful monotherapy in low-, intermediate-, and carefully selected high-risk localized prostate cancer [113–115]. In an investigation into the relationships between therapy and prostate cancer-specific mortality (PCSM) in elderly high-risk patients, Hoffman et al. observed that PCSM was lower in patients without preexisting or surgically corrected cardiovascular disease, particularly if they received antiandrogen therapy combined with IRT and EBRT [116].

Long-term results (>10 years) of IRT are good with no biochemical evidence of disease in 60–98% of patients, according to their risk category [117]. When local recurrences occur after EBRT or radical prostatectomy failure, IRT salvage treatment can be offered to selected cases as shown by evidence from a pooled-analysis study [118] and Niehoff et al. [119].

### 3.8. Skin Cancer

The increase in nonmelanoma skin cancer (NMSC) in European populations has become a major public health concern and will presumably impact greatly on healthcare costs [120, 121]. With basal cell cancer (BCC) accounting for 80–90% of NMSC, surgery is first-line treatment with RT as a valid alternative, particularly in elderly patients [122]. Although few studies have compared surgery and RT, a recent meta-analysis concluded that both are effective, safe, and associated with low recurrence rates [123]. In several studies HDR-IRT emerged as suitable one for elderly patients because of its short treatment time and low toxicity rates [124], achieving a 100% complete remission rate in a retrospective study of 20 patients with 23 lesions, with erythema being reported as the most frequent adverse event. Cosmesis was excellent as over 60% of cases were without skin alterations [124].

In another study, 11 patients, with a median age of 80 years, poor performance status, and scalp and face skin lesions, were treated with customized applicators. After a median follow-up of 16 months the 2-year local control rate was 91%, with no high-grade skin toxicity and only low-grade dermatitis (grade I: 50%, grade II: 33%) [125].

### 3.9. Breast Cancer

Approximately 45% of breast cancers develop in women aged >65 years and 33% in women aged >70 years [126, 127]. Although these data no studies have focused on elderly women who are often undertreated compared with younger counterparts [128, 129], consequently, the cancer mortality reduction cannot be compared [130, 131]. In a randomized clinical study, 636 women (age >70 years, clinical stage I, estrogen receptor (ER) positive, and breast carcinoma treated by lumpectomy) were assigned to Tamoxifen plus RT (317 women) or Tamoxifen alone (319 women). Median follow-up was 12.6 years. At 10 years, the disease-free local and regional recurrences were 98% in Tamoxifen and RT arm compared with 90% in the Tamoxifen alone group. No significant differences emerged in time to mastectomy or distant metastasis, breast cancer-specific survival, or OS [132].

In another randomized clinical trial, 1326 women, aged 65 years or older with low-risk early breast cancer who had had breast-conserving surgery and were receiving adjuvant endocrine treatment, were assigned to whole-breast RT (40–50 Gy in 15–25 fractions) or no RT. After a median follow-up of 5 years, ipsilateral breast tumor recurrence was 1.3% in women assigned to whole-breast RT and 4.1% in those who received none (*p* = 0.0002). No intergroup differences emerged in regional recurrence, distant metastases, contralateral breast cancers, or new breast cancers. The 5-year OS rate was 93.9% in both groups (*p* = 0.34) [133].

In 79 elderly low-risk breast cancer patients (median age 77 years; range 66–89), Sumodhee et al. [134] tested accelerated Partial Breast Irradiation (APBI) against whole-breast irradiation (WBI) or endocrine therapy alone. Median follow-up was 96.8 months (range 68.6–104.9). Expected 5- and 10-year mastectomy rates without WBI were 2.95% and 7.25%, respectively, leading to a 10-year metastasis-free survival (MFS) rate of 92.7%. Instead, expected 5- and 10-year mastectomy rates following WBI were 1.41% and 3.66%, respectively. The 10-year MFS rate after APBI was 97.4% versus 96.3% after WBI (*p* = 1) and 92.7% after no irradiation (*p* = 0.27). No grade 3 or grade 4 toxicity was observed.

Genebes et al. evaluated 70 APBI patients (median age 80.7 years; range: 62–93.1) with, respectively, 61.4%, 18.6%, and 20% classified as suitable, cautionary, and unsuitable cases according to American Society for Radiation Oncology (ASTRO) criteria. In interstitial multicatheter HDR-IRT, catheters were implanted intra- or postoperatively following lumpectomy and axillary sentinel lymph node dissection. Patients had a median follow-up of 60.9 months (range 4.6–90.1). The 5-year local recurrence-free rate was 97.6%. Five-year specific and overall survival rates were 97.9% and 93.2%, respectively. Late complications in 48% of patients encompassed grade 1 (80.8%) and grade 2 (19.2%) with no grade ≥3. Cosmetic results were considered excellent/good in 67 patients (95.7%) [135].

## 4. Conclusions

Evidence from this review shows that IRT therapy is of benefit to elderly patients with cancer, whatever its location or stage and whether IRT is used as monotherapy in combination with other agents or as palliative therapy. Treatment decisions in elderly cancer patients need to be taken by a multidisciplinary team, who carefully analyze tumor extent, the therapeutical alternatives, and the patient's preferences. Quality of life is probably a major outcome measure for the elderly as they value its preservation over receiving aggressive treatments with certain toxicity profiles. In terms of the patient's midterm quality of life, IRT is the optimal method for applying very high doses locally with maximal preservation of normal neighboring structures in the shortest total treatment time. Additional research into IRT is, however, needed, to strengthen the evidence for its use in elderly cancer patients [136, 137].

## Figures and Tables

**Table 1:** Comparison of different radiotherapy techniques regarding local dose application potentials.

	CK	IMRT	IGRT	IRT
Target definition	Worse^*∗*^	Worse^*∗*^	Worse^*∗*^	Better
Interfraction movements	Better	Worse	Better	Better
Intrafraction movements	Better	Better (4D)	Better (4D)	Better
Target dose painting	Better	Better (4D)	Better (4D)	Better
Low dose volumes	Worse	Worse	Worse	Better
OAR's dose	Better	Worse	Worse	Better
Invasivity	Better	Better	Better	Worse
Smallest reasonable CTV	0.5 cm^3^	>2 cm^3^	>2 cm^3^	0.5 cm^3^

CK = cyberknife; IMRT = Intensity Modulated Radiotherapy; IGRT = Image Guided Radiotherapy; IRT = interventional radiotherapy (brachytherapy); CTV = Clinical Target Volume; Worse^*∗*^ = turns into “better” if image fusion is used for treatment planning; 4D = four-dimensional external beam radiotherapy.
